# Oomycete transcriptomics database: A resource for oomycete transcriptomes

**DOI:** 10.1186/1471-2164-13-303

**Published:** 2012-07-06

**Authors:** Sucheta Tripathy, Tejal Deo, Brett M Tyler

**Affiliations:** 1 Virginia Bioinformatics Institute, Virginia Tech, Blacksburg, VA, 24061, USA; 2 Present address: The Realtime Group, 3035 W. 15th Street, Plano, TX, 75075, USA; 3 Present address: Center for Genome Research and Biocomputing, Oregon State University, Corvallis, OR, 97333, USA

**Keywords:** Transcriptomics, NGS methods, Database, Browser, Annotation

## Abstract

**Background:**

Oomycete pathogens have attracted significant attention in recent years due to their economic impact. With improving sequencing technologies, large amounts of oomycete transcriptomics data are now available which have great biological utility. A known bottleneck with next generation sequencing data however lies with their analysis, interpretation, organization, storage and visualization. A number of efforts have been made in this respect resulting in development of a myriad of resources. Most of the existing NGS browsers work as standalone applications that need processed data to be uploaded to the browser locally for visualization. At the same time, several oomycete EST databases such as PFGD, ESTAP and SPC, are not available anymore, so there is an immediate need for a database resource that can store and disseminate this legacy information in addition to NGS data.

**Description:**

Oomycetes Transcriptomics Database is an integrated transcriptome and EST data resource for oomycete pathogens. The database currently stores processed ABI SOLiD transcript sequences from *Phytophthora sojae* and its host soybean (*P. sojae* mycelia, healthy soybean and *P. sojae-*infected soybean) as well as Illumina transcript sequences from five *Hyaloperonospora arabidopsidis* libraries. In addition to those resources, it has also a complete set of Sanger EST sequences from *P. sojae*, *P. infestans* and *H. arabidopsidis* grown under various conditions. A web-based transcriptome browser was created for visualization of assembled transcripts, their mapping to the reference genome, expression profiling and depth of read coverage for particular locations on the genome. The transcriptome browser merges EST-derived contigs with NGS-derived assembled transcripts on the fly and displays the consensus. OTD possesses strong query features and the database interacts with the VBI Microbial Database as well as the *Phytophthora* Transcriptomics Database.

**Conclusion:**

Oomycete Transcriptomics Database provides access to NGS transcript and EST data for oomycete pathogens and soybean. The OTD browser is a light weight transcriptome browser that displays the raw read alignment as well as the transcript assembly and expression information quantitatively. The query features offer a wide variety of options including querying data from the VBI microbial database and the *Phytophthora* transcriptomics database. The database is publicly available at http://www.eumicrobedb.org/transcripts/.

## Background

Oomycete pathogens cause devastation to a wide range of hosts belonging to both plant and animal kingdoms [1]. Superficially, oomycete pathogens resemble fungi, but in fact they belong to a kingdom of life called Stramenopila, which also contains algae such as kelp and diatoms. Hence, conventional fungal control measures often fail against these pathogens [2]. *Phytophthora* species and many other members of the order Peronosporales cause destructive diseases in an enormous variety of crop plant species as well as forests and native ecosystems [3]. The potato pathogen, *P. infestans*, was responsible for the Irish potato famine and is still a destructive pathogen of concern for bio-security [4]. In the past few years, whole genomes, transcriptomes and ESTs have been sequenced for many oomycete species [1,5-7]. With the rapid growth of next generation sequencing (NGS) technologies such as those of 454 Life Sciences, Illumina and ABI SOLiD, informatics tools and resources have increasingly become a bottleneck. Several oomycete EST databases described previously are no longer available, including the Syngenta *Phytophthora* Consortium (SPC) EST sequence data bases at https://xgi.ncgr.org/spc; the *Phytophthora* Functional Genomics Database at http://www.pfgd.org[8]; and ESTAP (EST Analysis Pipeline) [9] at http://staff.vbi.vt.edu/estap/. Recently a new transcriptomics database, *Phytophthora* transcriptomics database (PTD) was created at Nanjing Agricultural University, that contains digital gene expression information from *Phytophthora sojae*[10]. We previously created the VBI microbial database (VMD) [11], that served as a data warehouse for several oomycete genome sequences. However, the schema level of VMD did not readily accommodate NGS transcriptomic data. Therefore, we have created the Oomycete Transcriptomics Database (OTD) to store oomycete transcriptomics data and easily interface with VMD and PTD.

One challenging feature of presenting transcriptomics data produced by next generation sequencing (NGS) methods is data visualization on a browser. Most of the existing NGS browsers are stand alone applications that require users to upload processed data into the browser for visualization. This is a significant drawback, since the users must have access to the processed information or else they need to run analysis pipelines. We have created a web based transcript browser that displays EST transcripts, NGS transcripts, their alignment to the reference genome, genome annotation features and merged EST and NGS transcripts.

## Construction and contents

OTD is a relational database with a backend that uses MySQL version 5.1.49, a front end that uses PHP version 5.3.3 and PERL CGI version 5.12.1. All the visualization tools were created using PERL GD, GnuPlotter and Image Magik.

The *P. sojae* ABI SOLiD sequences were obtained from mycelial transcripts and from soybean hypocotyls 12 h after inoculation with *P. sojae* (four replicates each). The Soybean ABI SOLiD sequences were obtained from the mock inoculated transcripts and 12 h *P. sojae* post infected samples. Details of the production of these data will be published elsewhere. The *H. arabidopsidis* Illumina reads were obtained from Arabidopsis leaves 7 days after inoculation with the pathogen [7]. The EST sequences of *P. sojae* were generated from six different cDNA libraries [12] and the *P. infestans* EST sequences, downloaded from Genbank [13].

### Data processing

Raw SOLiD, Illumina and EST sequences were preprocessed and analyzed prior to uploading them into the database.

#### Processing of next generation sequencing data

**Figure 1:**
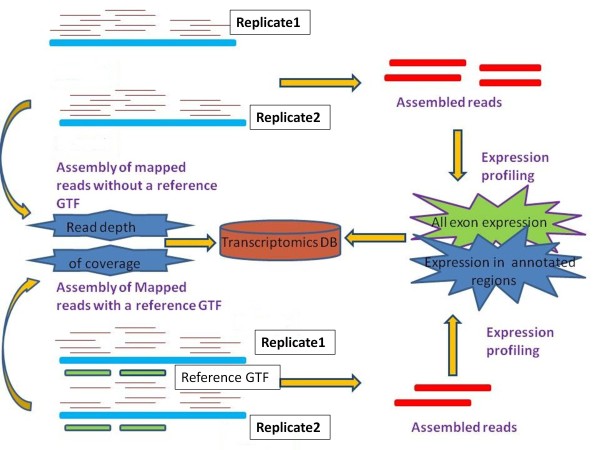
**Workflow diagram describing generation of assembled transcripts from RNAseq data.** Data from a single treatment with different replicates (1,2,3,4) merged together and assembled with Cufflink using 2 different options: a. With a reference annotation file (with GTF) b. Without a reference annotation file (without GTF). With-GTF only provides assembly where annotations are available whereas without-GTF provides extra annotation information. Outputs from these 2 options are merged and stored in the database.

We worked with approximately 63 million SOLiD reads from the *P. sojae* mycelial library, 240 million reads from the *P. sojae* soybean infection library and 900 million Soybean mock inoculated libraries with an average read length of 50 bases. We included around 6 million cDNA Illumina reads (210 MB) from 5 replicates of *H. arabidopsidis*-infected Arabidopsis leaf samples 7 days post-inoculation [7]; these were downloaded from an EBI repository http://www.ebi.ac.uk/ena/data/view/ERP000272. We aligned all the NGS reads from *P. sojae* and *H. arabidopsidis* with their respective genome assemblies using BowTie [14]. For *P. sojae,* we used two different genome assembly versions v1.0 and v5.0 (to be described elsewhere) whereas for *H. arabidopsidis* we used the latest assembly version 8.3.2 [7] [Table 1. For *P. sojae* mycelial data, only about 50% of the reads found a match with the reference in V1.0 genome assembly, while this number was slightly greater (about 55%) with V5.0 assembly. For *P. sojae* infection samples, the number was poor for both the assembly versions. The read depth of coverage was calculated for each nucleotide of each genome, if at least one read from each replicate spanned it. 13.5 million bases of the *P. sojae* genome were represented by at least one read from each of the four mycelial replicates and 15.1 million bases were represented by at least one read from each of the infection library replicates. Four million bases from the *H. arabidopsidis* version 8.3 assembly were represented by the Illumina reads. Read quantification and transcript assembly were computed using CuffLink [15]. CuffLink detected many highly expressed exons that were not included in the present genome annotation [5,7]. We used the CuffCompare utility (a part of the CuffLink distribution) to merge the expression data across different libraries. CuffCompare was run with and without the genome annotation option, so that we could curate and compare the expression results for annotated genes and the novel exons [Figure 1.

**Table 1:** RNAseq read alignment statistics

**NGS library**		***P. sojae *mycelia (4 replicates)**				***P. sojae*-infected soybean (4 replicates)**			
***P. sojae***		**PS1**	**PS2**	**PS3**	**PS4**	**WI1**	**WI2**	**WI3**	**WI4**
assembly	v1.0	8491356	8569508	14643646	4748944	8645377	10382780	7331138	7272520
		(54.2%)*	51.4%	(50.8%)	(37.6%)	(13.7%)	(17.5%)	(11.1%)	(13.1%)
	v5.0	8909517	8924547	15248004	5141252	6862159	8978294	7067909	6713689
		(56.9%)	(53.5%)	(52.9%)	(40.7%)	(10.8%)	(15.1%)	(10.7%)	(12.1%)
NGS library		*H. arabidopsidis*-infected *Arabidopsis* (5 replicates)							
*H. arabidopsidis*		sample 1	sample 2	sample 3	sample 4	sample 5			
assembly	v8.3	247198	72427	82827	295351	517242			
		(21.4%)	(16.8%)	(16.4%)	(28.7%)	(26.6%)			

*Numbers and percentages of RNAseq reads aligned with the assembled reference genome.

We carried out a *de novo* assembly of the reads to generate contigs using the Abyss assembler [16]. The assembled contigs were then mapped onto the genome assembly using BLAT [17]. A number of assembled contigs that did not match with the genome assembly were annotated and stored in the database.

Cufflink-based transcript assembly requires a reference for assembly. So, we assembled the transcripts from mycelia and infection libraries against two different reference genome assembly versions from *P. sojae*. We then carried out an overlap analysis between these assembled transcripts and the existing EST data and gene model data [Table 2. The number of assembled transcripts without any EST or gene model support was slightly higher in *P. sojae* V1.0 (22%) than in *P sojae* V5.0 (18%). The percentage of transcript assemblies not covered by predicted gene models in assembly version 1.0 was consistently higher (30%) than assembly version 5.0 (23%). The percentages of transcript assemblies covered by both gene models and ESTs were about 26% for both the genome assembly versions. For the *H. arabidopsidis* transcriptome, the percentages of transcripts overlapping with predicted gene models and ESTs were even less [7].

**Table 2:** Assembled Transcripts Supported by Predicted models and EST libraries

**Library/Assembly Version**	**Version1.0**	**Version5.0**	**Version1.0**	**Version5.0**
	**Mycelial**	**Mycelial**	**Infection**	**Infection**
Total Number of assembled Transcripts	26,742	28,955	30,522	31,239
Number of Transcripts with both EST and	6,967	7,544	7,714	8,179
Gene Model support	(26%)	(26%)	(25%)	(26%)
Number of Transcripts with no match to	8,078	6,887	8,732	7,126
Gene Models	(30%)	(23.78%)	(28.6%)	(22.8%)
Number of Transcripts with No match to	17,696	19,789	20,598	21,371
EST sequences	(66%)	(68%)	(67%)	(68%)
Number of Transcripts having no match to	5,999	5,265	6,522	5,437
gene models or ESTs	(22%)	(18%)	(21%)	(17.4%)

#### EST sequence preparation and processing

The raw *P. sojae* and *H. arabidopsidis* EST sequences were obtained as chromatograms. The sequence files and qual files were extracted using PHRED [18] with a command line option –trim_alt and a cutoff parameter of 0.1. As part of the cleaning protocol, the sequences were quality trimmed using an in-house algorithm. The maximum number of low-quality bases (quality score < 20 for 5′ end and < 15 for 3′ end) allowed in a window of size 25 was 6 for both the 3′ and 5′ end. Windows having > 6 low-quality bases were shifted one base, and the process was repeated.

For vector removal, CrossMatch [19] was used with the –minmatch and –minscore parameters set to 10 and 20, respectively. For adaptor removal, both these parameters were lowered to 8, so that smaller adaptors could be removed. Internal poly A/T tracts (indicating chimeric cDNA fragments) were removed and the sequence cleaved if the tract length was > 18 bases. For terminal poly A/T tracts, the tract length parameter was removed.

Contaminating sequences with very strong (95%) similarity with vector or any other sequence database were removed prior to clustering. The ESTs from infection libraries were initially assigned to host or pathogen by the procedure (Additional file 1: Figure S1). Later when the genome sequences of the pathogen and host became available, the assignments were checked and if necessary corrected. The soybean ESTs recovered from the analysis of the infection libraries were submitted to GenBank and can be found with accession numbers between CF805618-CF809370.

The clean EST sequences were clustered and assembled using the TGICL wrapper [20]. TGICL uses megablast [21] for clustering and CAP3 [22] for assembly. The analysis was run on a Sun server with 2 Xeon 3-GHZ processors and 4 GB RAM with Slackware Linux (i486). The minimum percent of identity for overlaps was kept at 94, minimum overlap length was kept at 30, and maximum length of unmatched overhangs was kept at 30 for CAP3 alignment. Finally, 7,863 unigenes from *P. sojae*, 2,292 unigenes from soybean (derived from *P. sojae*-infected tissue), 14,754 unigenes from *P. infestans* and 13,363 unigenes from *H. arabidopsidis* were obtained.

#### Identifying protein coding regions

We identified the protein coding sequences from the unigenes using a modified log-likelihood algorithm [23]. This algorithm calculates the coding potential across a sliding window of user-defined size (we used 120) for all six frames and determines the most likely coding frame. Then it compares the islands with higher coding potential with known sequence patterns such as start and stop codons. If the start/stop pattern is found around the window size where there is high coding potential, then that region is called a coding sequence. Cases of frame shift sequence errors, chimeric sequences and contamination were easily detected using this algorithm and marked accordingly. Once protein coding regions were marked, the sequence annotation steps required much less processing time.

#### Alignment of assembled EST unigenes to genome sequences

The unigenes derived from EST sequences of *P. sojaeP. infestans* and *H. arabidopsidis* were aligned to their respective genome assemblies as well as to other oomycete genome assemblies using BLAT [17]. All the alignments were carried out with a minimum alignment ratio of 0.93, and the minimum size of alignment of 20. The alignments were ranked from 1 to 4 using the following criteria:

1) More than 95% identity and no query gaps.

2) More than 95% identity. Query gaps exist and can be explained by the presence of plausible genomic sequence gaps.

3) More than 95% identity. Query gaps exist that can’t be explained by genomic sequence gaps but are less than 10 bases and/or end mismatches are present but are less than 10 bases.

4) More than 95% identity. Query gaps that can’t be explained by genomic sequence gaps are more than 10 bases and/or end mismatches are present and more than 10 bases.

#### Annotation

The primary annotation of the sequences was done with tera-BLASTX against a non-redundant protein database accelerated on the TimeLogic’s DeCypher system. The Blast outputs were parsed, and up to 10 significant blast hits with associated HSP data were stored in the database. For functional annotation of the protein sequences, we used InterProScan [24]. We sent smaller chunks of sequences to the server to optimize the resource usage. The data were parsed and stored in the database. Secretory and membrane proteins were predicted by running signalP [25] and TMHMM [26] on the protein sequences. The annotations are updated every six months. The last time annotations were updated was during Nov 2011.

### Database design and creation of user interface

OTD features multiple data types such as raw NGS reads, assembled reads, raw ESTs, assembled ESTs and their annotation and mapping to reference genomes. A brief Entity relationship diagram of the database is provided (Additional file 2: Figure S2). The database stores the following information:

1) Read depth of coverage of NGS data

2) Assembled reads generated from mapped and unmapped NGS reads

3) Levels of existing and novel transcripts expressed as FPKM [Fragments Per Kilobase of exons per Million fragments mapped]

4) Cleaning, clustering, assembly, and annotation information for EST data.

## Utility and discussion

### Transcriptomics browser

**Figure 2:**
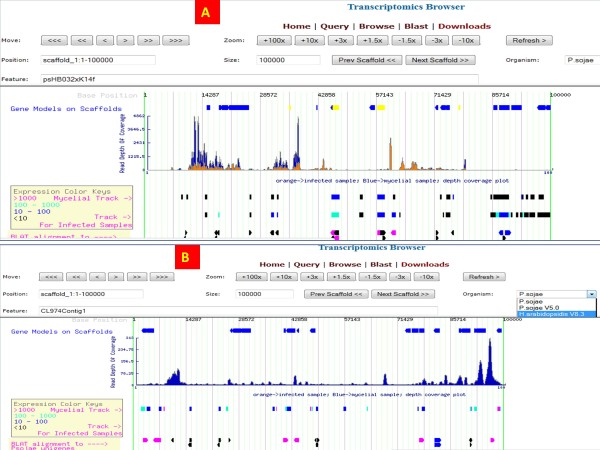
**Screenshots of Transcriptomics browser.** (**A**) Transcripts page for *P.sojae* V1.0 assembly. (**B**) *H. arabidopsidis* 8.3 assembly.

We have created a new web based browser for visualization of NGS data. The browser retrieves genomic features such as predicted gene models and their annotations from VMD [11], and retrieves the transcriptome information from OTD. The first track on the browser displays the gene models predicted from the genomes, followed by the transcript depth of coverage track. The transcript depth of coverage is plotted in two different colors e.g. yellow for infected samples and blue for mycelia samples for *P. sojae*. For *H. arabidopsidis*, the track just displays one color, orange since the samples are only from infection library. The next two tracks are for transcript assembly where the transcripts are color coded for their expression values calculated in FPKM. Transcripts with expression values > 1000 FPKM are considered very highly expressed and are color coded in red; transcripts with moderate to high levels of expression (FPKM value between 100 and 1000) are coded in green; transcripts with low to moderate levels of expression (between 10 and 100) values are represented in blue; low expressed transcripts (< 10) are represented in black [Figure 2A, B]. The remaining tracks are the EST-derived unigenes mapped into the reference genome assembly. These are also color-coded according to the quality of alignment to the genome sequence and the coloring scheme is similar to that of Genbank blast results. The best alignments, in which there are no gaps in the query or subject alignments are color coded in red; the next best alignments, that have subject gaps but no query gaps are coded in green; the third category, in which there are both query gaps and subject gaps are coded in blue and the poorest category, that contains query gaps as well as mismatches, is coded in black.

The transcriptomics browser is the central component of the resource that enables users to walk over the genome assembly and discover important transcribed elements that may be missing from the annotation. One can switch from one organism to another on the browser by selecting the organism from the top panel drop down box of the main transcriptomics browser page [Figure 2A, B].

**Figure 3:**
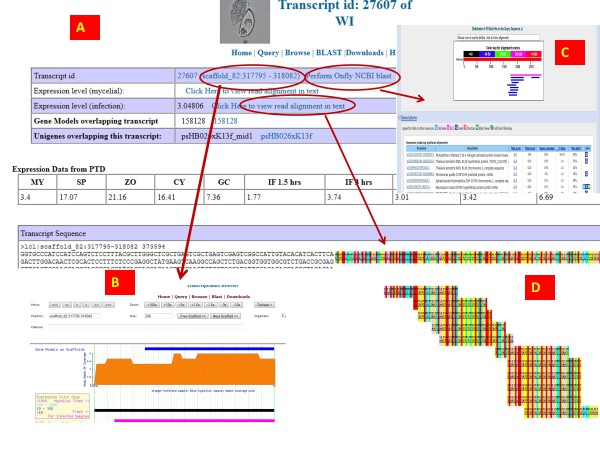
**(A) Screenshot of transcript assembly page.** The circled area representing the genomic location of the transcript links into the transcript browser (**B**). The on-the-fly BLAST link carries out a nr blast against Genbank (**C**). The reads assembly link opens to the SNP viewer (**D**). Note the SNP viewer has the reference genome sequence on top which is static on the screen and as one scrolls down, it follows along, so that users can always overlap transcript sequences against the reference genome.

**Figure 4:**
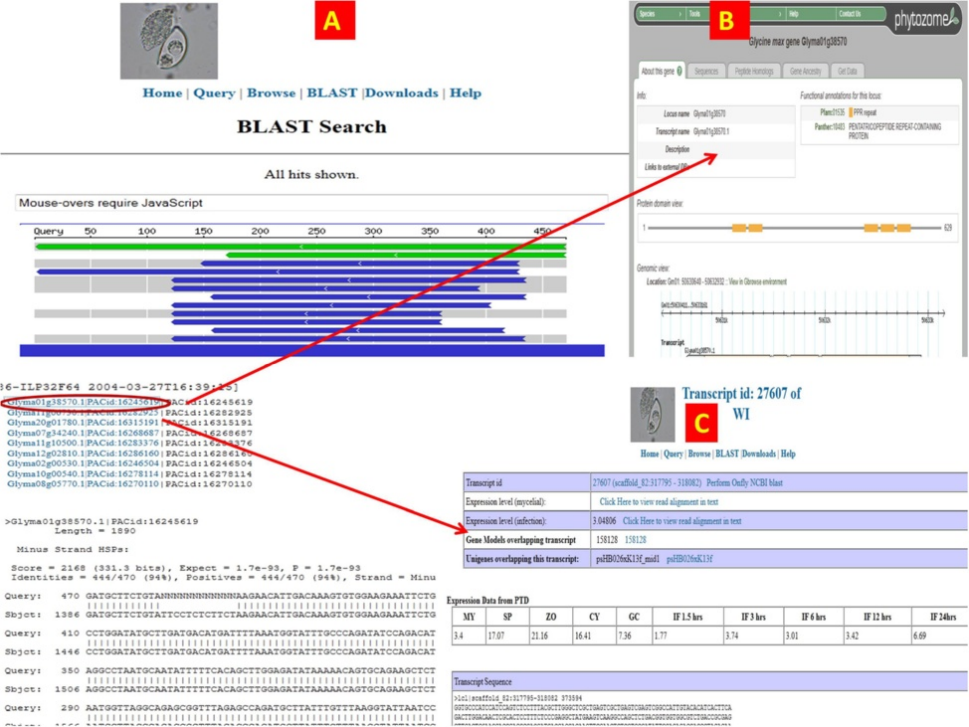
**Screenshot of the SNP viewer. In this view, three different screens are merged.** The first view (**A**) indicates there are some polymorphisms with the reads. The reference sequence remains fixed to the top of the browser window. As the user scrolls down (**B, C**), the reference sequence scrolls down along and the read bases can be super-imposed with the reference for detecting SNPs and finding intron-exon boundaries.

All the tracks are clickable leading to the transcript assembly page or EST unigene page depending on the tracks (more details in Additional file 3). The transcript assembly page contains extensive information starting with the location of the transcript on the genome. If the transcript overlaps with ESTs or predicted gene models, links to the EST and gene model page is provided [Figure 3A]. From the scaffold location link, one can reach the transcriptomics browser [Figure 3B]. Recently, we have mirrored data from PTD, which is displayed on the main transcript page. On-the-fly Genbank blast feature is available from main transcript page [Figure 3C]. The reads assembly and SNP viewer are linked from the main transcript page [Figures 3D, 4]

### Web based reads alignment viewer or SNP viewer

We have created a web based text alignment viewer on the reference genome. This viewer can also be used for SNP viewing and for correcting gene models based on the alignment of the transcript reads to the reference genome. Links to the text based viewer are provided from the main transcript assembly page that is based on the reads assembly on the reference strand. The top most row is the genomic reference followed by the reads mapped to them arranged in rows. As the number of reads increase, the page needs to be scrolled down and towards right to view the alignment. We have used java script for fixing the position of the reference strand on the screen vertically, so the users can always superimpose reference bases with the read bases (Figure 4). This greatly helps in detecting substitutions, intron—exon location and a false assembly.

### Transcriptomics blast site

**Figure 5:**
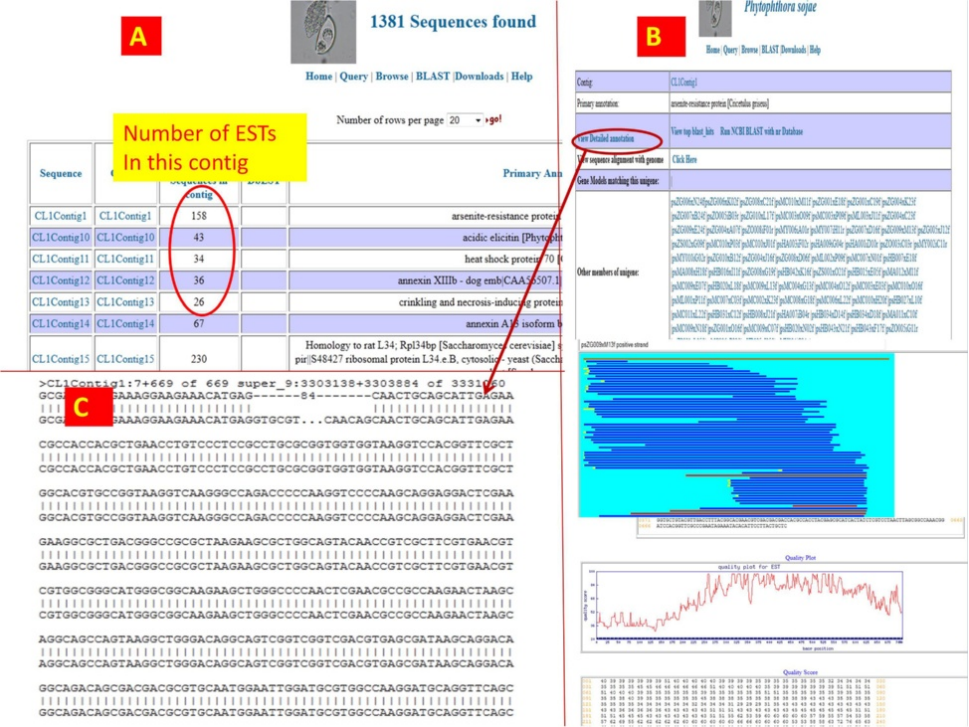
**Screenshot of transcriptomics Blast page and output pages.** The blast output page against a query sequence opens into (**A**). The subject sequences from the blast output provide links to the appropriate databases. For example, if the data has additional relevant information in Phytozome, then the link opens to the Phytozome website (**B**). If the data is present internally then the links open to OTD (C).

We have significantly upgraded the transcriptomics Blast utility which carries out Wublast [27] against 23 transcriptomics databases. The graphical user interface of the blast utility uses the standard bioperl Bio::GMOD::Blast::Graph package. We replaced the HTML writer utility with our own perl package, so that subject values would point to the correct links in our database [Figure 5B, C]. All of the transcripts assembled from the NGS reads along with several additional datasets such as Soybean CDS are available for blast. If the user chooses external databases such as the soybean genome and Soybean predicted transcripts, the links are directed towards the Phytozome web site [Figure 5B]. For the internal databases such as the transcript assembly database, the link directs to the main transcript page in OTD [Figure 5C. If an EST or inhouse database is searched against, then the link directs to the appropriate page.

### Main annotation page for unigenes

**Figure 6:**
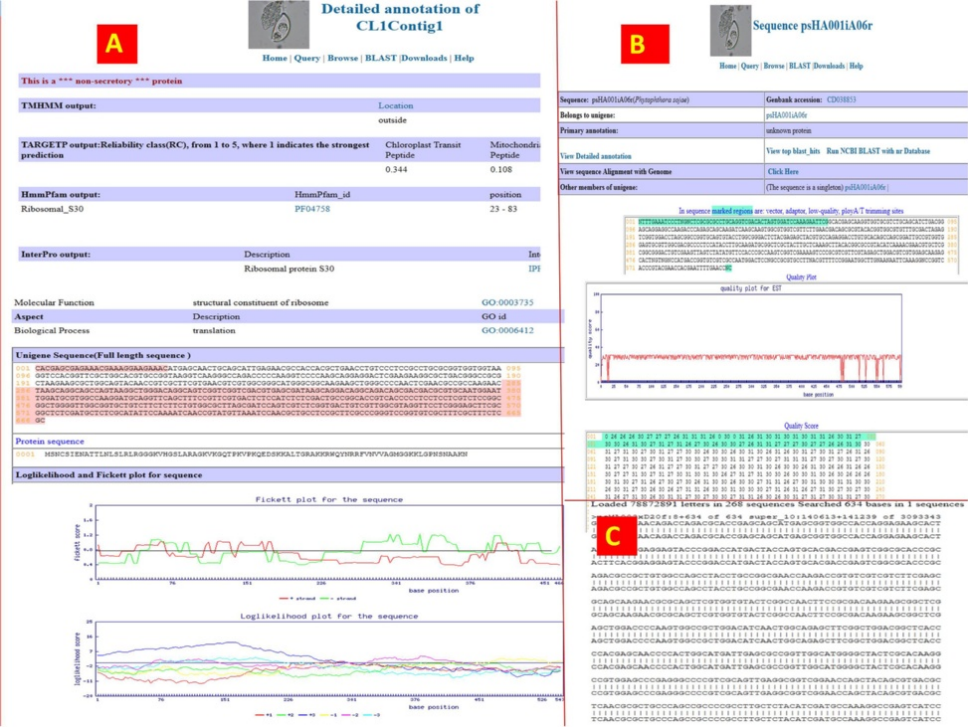
**Screenshots illustrating query by unigene name.** Results shown are from a wild card search with “CL1C.*”. (**A**) The output of the search. Click on the contigs (second column) links to the unigene or contig page (**B**) that has several information items such as the constituent ESTs, functional annotation, on-the-fly blast and an on-the-fly option for BLAT against the reference genome (**C**).

**Figure 7:**
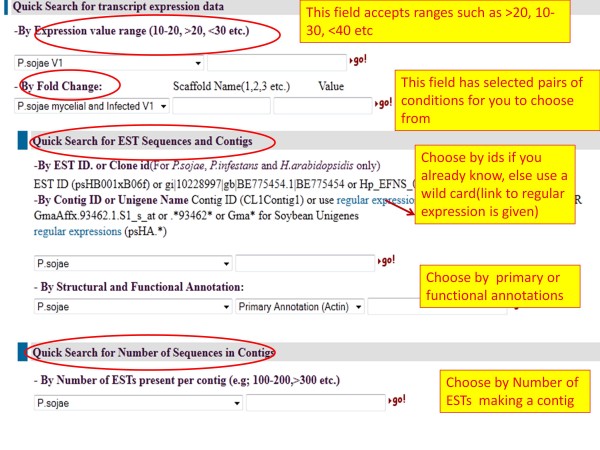
**Main Contig/Unigene annotation page.** From the contig page described in Figure 6A, there is a link to the detailed annotation page (**A**). There are several annotations available such as interproscan, TMHMM, SignalP etc. We used an in-house program to predict UTRs in unigenes. The highlighted areas in (**A**) are the UTRs. Further down on the page, there are Fickett statistics and a log-likelihood plot for that sequence confirming presence of UTRs. (**B**) Shows the annotation page for an EST singleton. The highlighted area contains the EST trimming information and below the sequence there is a base quality plot. (**C**) Shows on-the-fly blat result of the EST against the genome.

Each unigene whether derived from ABI SOLiD, Illumina or EST data is given a unique id and has a primary annotation page and a detailed annotation page. The primary annotation page includes component ESTs that make up the unigenes. Unigenes can be queried by name from the query page with a wild card search or an absolute string search. If a wildcard search is performed, then a number of unigenes will be displayed on the output page, with a lot of information such as the number of component ESTs making up that unigene, their primary annotations etc. [Figure 6A]. On click, each unigene page opens onto a new page that lists basic information about the unigene, the assembly plot, the primary annotation, links to the unigene annotation detail page etc. [Figure 6B]. The assembly plot of the component EST sequences displays the matching and non-matching regions in a sequence cluster. This helps users judge the quality of the assembly. From the unigene primary annotation page, a one-click link is provided for BLAST searches against the NCBI nr database. If the unigene has an overlap with a gene model predicted from the genome sequence, then a link to the gene is provided on the primary annotation page. Also, users can choose to run a BLAT alignment of the unigene against the reference genome on-the-fly [Figure 6C]. The detailed annotation pages for unigenes and contigs have details on InterProScan, TMHMM and SignalP annotations, and coding frame and ORF information [Figure 7A].

Each component EST sequence of a unigene, if present is provided with a link, so that the user can reach the EST details with a click. The individual EST page has quality trimming protocols, other ESTs that overlap with the sequence and many more relevant information [Figure 7B]. Also an on-the-fly BLAT option is available for EST sequences against the respective reference genome [Figure 7C].

### Query page

**Figure 8:**
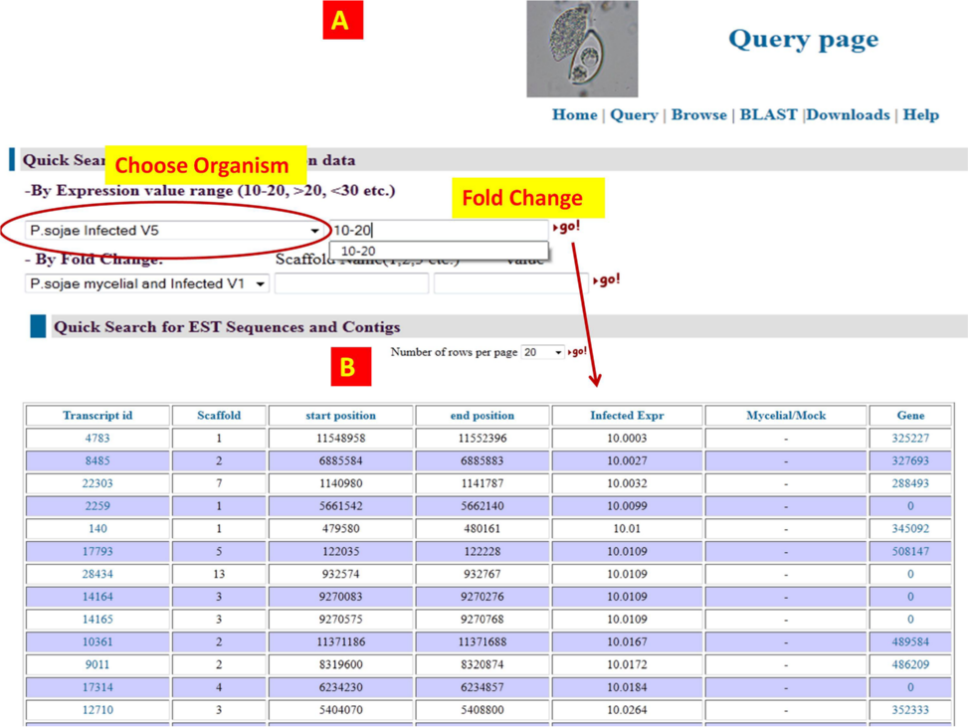
Screenshot of the query page.

A user-friendly query page enables users to query OTD using the following categories [Figure 8]:

1. By fold change in treated versus untreated samples.

2. By expression value.

3. By names of the unigenes or ESTs or contigs.

4. By primary and secondary annotation.

5. By number of ESTs present in a unigene.

**Figure 9:**
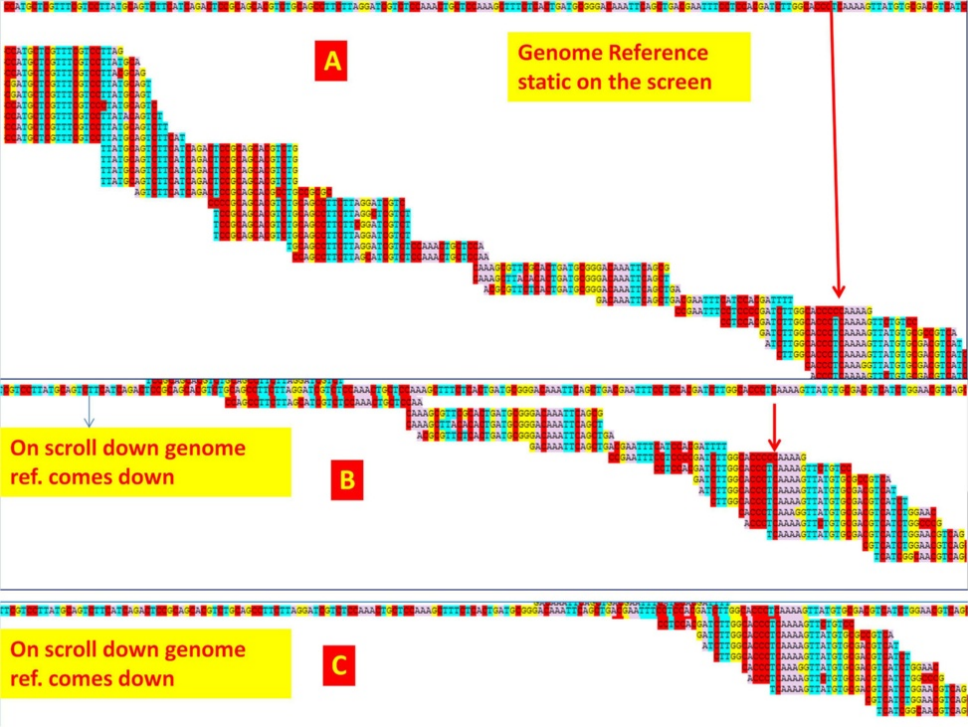
**Screenshot of query by expression value.** In this example, an expression value between 10–20 was chosen for genes from *P. sojae* V5.0 assembly (**A**). The search retrieves a number of records (**B**), where the first column contains the links to the assembled transcript_ids. The last column represents the overlapping gene models, EST sequences etc.

Expression values of transcripts are represented as FPKM values. Users can choose an absolute value or a value range such as 10–20, <10, > 10 etc. to query the database. If a range value is chosen, then a number of records are retrieved and displayed on a page. Links are provided from this page to go to individual transcript pages or the page for an overlapping EST or gene model (if available) in VMD [Figure 9A, B].

Another useful query feature is that ability to retrieve transcripts that have a fold change between two given conditions. For example, in the case of the *P. sojae* V1.0 assembly, one can query and find all the transcripts that show a certain fold change (e.g. two-fold) between infection and non-infection conditions. Similar search options are also available for soybean datasets. Due to the data size and query time, options are currently restricted to searching by individual scaffolds.

EST-derived unigenes and contigs can be searched by exact id name or by a regular expression. For example, most of the EST contigs begin with CL1, so, users can query the database with CL1* [Figure 6A]. If the user chooses to query by a single contig name, then the primary contig page with primary annotation and quality scores are displayed. If a contig has a overlapping gene model, the gene_id along with its VMD link is provided.

### Miscellaneous features

In addition to the utilities described above, there are a number of miscellaneous items available from the home page. Sequence statistics, cluster statistics, metadata information and library construction methods are accessible from this page. For *P. sojae* EST datasets, cluster statistics and details of the sequence distribution in EST clusters are listed with proper links to the main annotation pages.

The download site currently provides 39 curated data types for download. Users can request additional information if necessary through the available requisition form provided in the page.

## Conclusion

OTD, with its numerous visualization tools and backend processing pipelines, is a valuable resource for the oomycete community to browse and retrieve transcriptomics information. OTD is also linked with VMD and PTD for additional information on genome sequences and expression data. As additional genome and transcriptome data become available, they will be imported into the database.

## Availability and requirements

The database is publicly available at http://www.EuMicrobeDB.org/transcripts. The database and associated software are open source and will be made available upon request.

## Abbreviations

EST: Expressed sequence tags; ORF: Open reading frames; UTR: Un-translated regions; HSP: High-scoring Segment Pair; Qual: Quality score files; OTD: Oomycete Transcriptomics database; FPKM: Fragments per Kilobase of exons per Million fragments mapped; NGS: Next generation sequencing data; ABI: Applied Biosystems; PTD: Phytophthora Transcriptomics Database; VMD: VBI Microbial Database.

## Competing interests

The authors declare that they have no competing interests.

## Authors’ contributions

ST analyzed and uploaded the data, designed and created the database, browser, visualization tools and front end features. TD designed the user interface and schema and contributed to writing queries. BMT mentored and supervised the whole project and helped with annotation. ST and BMT wrote the manuscript. All authors read and approved the final manuscript.

## Supplementary Material

Additional file 1 **Figure S1.** Soybean Sequence filtration protocol from*P. sojae* EST libraries. The Uncertain sequences were manually assigned to *P. sojae* or *Soybean* and are stored in the database. Click here for file

Additional file 2 **Figure S2.** Entity-Relationship diagram of OTD. Click here for file

Additional file 3 Documentation on how to use the oomycete transcriptomics database. Click here for file

## References

[B1] Haas BJ Kamoun S Zody MC Jiang RH Handsaker RE Cano LM Grabherr M Kodira CD Raffaele S Torto-Alalibo T Genome sequence and analysis of the Irish potato famine pathogen Phytophthora infestans Nature 2009 461 7262 393 398 10.1038/nature08358 19741609

[B2] Gaulin E Bottin A Dumas B Sterol biosynthesis in oomycete pathogens Plant Signal Behav 2010 5 3 3 10.4161/psb.5.3.10551 PMC2881271 20023385

[B3] Erwin DC Ribeiro OK Phytophthora Diseases Worldwide: St Paul 1996 MN, USA: The American Phytopathological Society

[B4] Fry W Phytophthora infestans: the plant (and R gene) destroyer Mol Plant Pathol 2008 9 3 18 10.1111/j.1364-3703.2007.00465.x PMC6640234 18705878

[B5] Tyler BM Tripathy S Zhang X Dehal P Jiang RHY Aerts A Arredondo FD Baxter L Bensasson D Beynon JL Phytophthora genome sequences uncover evolutionary origins and mechanisms of pathogenesis Science 2006 313 5791 1261 1266 10.1126/science1128796 16946064

[B6] Lévesque CA Brouwer H Cano L Hamilton JP Holt C Huitema E Raffaele S Robideau GP Thines M Win J Genome sequence of the necrotrophic plant pathogen Pythium ultimum reveals original pathogenicity mechanisms and effector repertoire Genome Biol 2010 11 7 R73 10.1186/gb-2010-11-7-r73 20626842 PMC2926784

[B7] Baxter L Tripathy S Ishaque N Signatures of adaptation to obligate biotrophy in the Hyaloperonospora arabidopsidis genome Science 2010 330 6010 3 10.1126/science.1195203 PMC3971456 21148394

[B8] Gajendran K Gonzales MD Farmer A Archuleta E Win J Waugh ME Kamoun S Phytophthora functional genomics database (PFGD): functional genomics of Phytophthora-plant interactions Nucleic Acids Res 2006 34 5 10.1093/nar/gnj007 PMC1347481 16381913

[B9] Mao C Cushman JC May GD Weller JW ESTAP–an automated system for the analysis of EST data Bioinformatics 2003 19 13 2 15593407 10.1093/bioinformatics/btg205

[B10] Ye W Wang X Tao K Lu Y Dai T Dong S Dou D Gijzen M Wang Y Digital gene expression profiling of the Phytophthora sojae transcriptome MPMI 2011 1530 9 10.1094/MPMI-05-11-0106 21848399

[B11] Tripathy S Pandey VN Fang B Salas F Tyler BM VMD a community annotation database for oomycetes and microbial genomes Nucleic Acids Res 2006 34 Database issue D379 D381 10.1093/nar/gkj042 16381891 PMC1347405

[B12] Torto-Alalibo TA Tripathy S Smith BM Arredondo FD Zhou L Li H Chibucos MC Qutob D Gijzen M Mao C Expressed sequence tags from Phytophthora sojae reveal genes specific to development and infection Mol Plant Microbe Interact 2007 20 7 781 793 10.1094/MPMI-19-1302 17601166

[B13] Randall TA Dwyer RA Huitema E Beyer K Cvitanich C Kelkar H Ah Fong AMV Gates K Roberts S Yatzkan E Large-Scale Gene Discovery in the Oomycete Phytophthora infestans Reveals Likely Components of Phytopathogenicity Shared with True Fungi MPMI 2005 18 3 15 15782637 10.1094/MPMI-18-0229

[B14] Langmead B Trapnell C Pop M Salzberg SL Ultrafast and memory-efficient alignment of short DNA sequences to the human genome Genome Biol 2009 10 R25 10.1186/gb-2009-10-3-r25 19261174 PMC2690996

[B15] Trapnell C Williams B Pertea G Mortazavi A Kwan G van Baren M Salzberg S Wold B Pachter L Transcript assembly and quantification by RNA-Seq reveals unannotated transcripts and isoform switching during cell differentiation Nat Biotechnol 2010 28 5 511 515 10.1038/nbt.1621 20436464 PMC3146043

[B16] Simpson JT Wong K Jackman SD Schein JE Jones SJM Birol İ ABySS: A parallel assembler for short read sequence data Genome Res 2009 19 6 7 10.1101/gr.089532.108 PMC2694472 19251739

[B17] Kent WJ BLAT–the BLAST-like alignment tool Genome Res 2002 12 4 9 10.1101/gr.229202 PMC187518 11932250

[B18] Ewing B Hillier L Wendl MC Green P Base-calling of automated sequencer traces using phred. I. Accuracy assessment Genome Res 1998 8 3 175 185 9521921 10.1101/gr.8.3.175

[B19] Ewing B Green P Base-calling of automated sequencer traces using phred. II. Error probabilities Genome Res 1998 8 3 186 194 9521922

[B20] Pertea G Huang X Liang F Antonescu V Sultana R Karamycheva S Lee Y White J Cheung F PBe: TIGR Gene Indices clustering tools (TGICL): a software system for fast clustering of large EST datasets Bioinformatics 2003 19 5 2 12651724 10.1093/bioinformatics/btg034

[B21] Zhang Z Schwartz S Wagner L Miller W A greedy algorithm for aligning DNA sequences J Comput Biol 2000 7 1–2 12 10.1089/10665270050081478 10890397

[B22] Huang X Madan A CAP3: A DNA sequence assembly program Genome Res 1999 9 9 10 10.1101/gr.9.9.868 PMC310812 10508846

[B23] McLachlan AD Staden R Boswell DR A method for measuring the non-random bias of a codon usage table Nucleic Acids Res 1984 12 24 9 10.1093/nar/12.24.9567 PMC320481 6393058

[B24] Zdobnov EM Apweiler R InterProScan–an integration platform for the signature-recognition methods in InterPro Bioinformatics 2001 17 9 2 10.1093/bioinformatics/17.9.847 11590104

[B25] Bendtsen JD Nielsen H von Heijne G Brunak S Improved prediction of signal peptides: SignalP 3.0 J Mol Biol 2004 340 4 13 10.1016/j.jmb.2004.05.028 15223320

[B26] Krogh A Larsson B von Heijne G Sonnhammer EL Predicting transmembrane protein topology with a hidden Markov model: application to complete genomes J Mol Biol 2001 305 3 14 10.1006/jmbi.2000.4315 11152613

[B27] Lopez R Silventoinen V Robinson S Kibria A Gish W WU-Blast2 server at the European Bioinformatics Institute Nucleic Acids Res 2003 31 13 4 10.1093/nar/gkg573 PMC168979 12824421

