# Supplementary figures and images for: Oomycete transcriptomics database: A resource for oomycete transcriptomes
Source: BMC Genomics. 2012 Jul 6;13:303. doi: 10.1186/1471-2164-13-303 (PMC3542173; doi:10.1186/1471-2164-13-303)

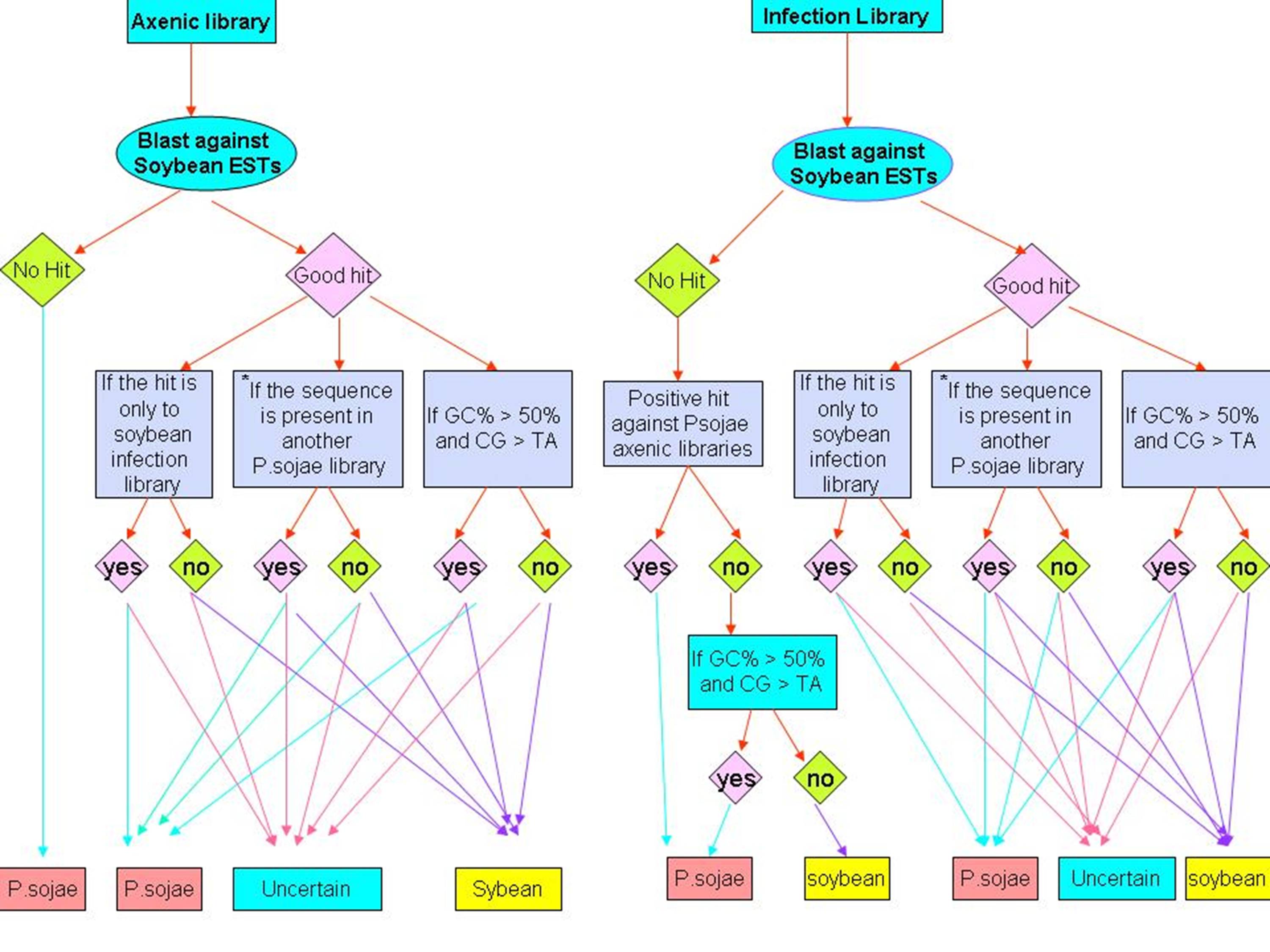

Supplement: Additional file 1 — Figure S1. Soybean Sequence filtration protocol fromP. sojae EST libraries. The Uncertain sequences were manually assigned to P. sojae or Soybean and are stored in the database. [file 1471-2164-13-303-S1.jpeg]

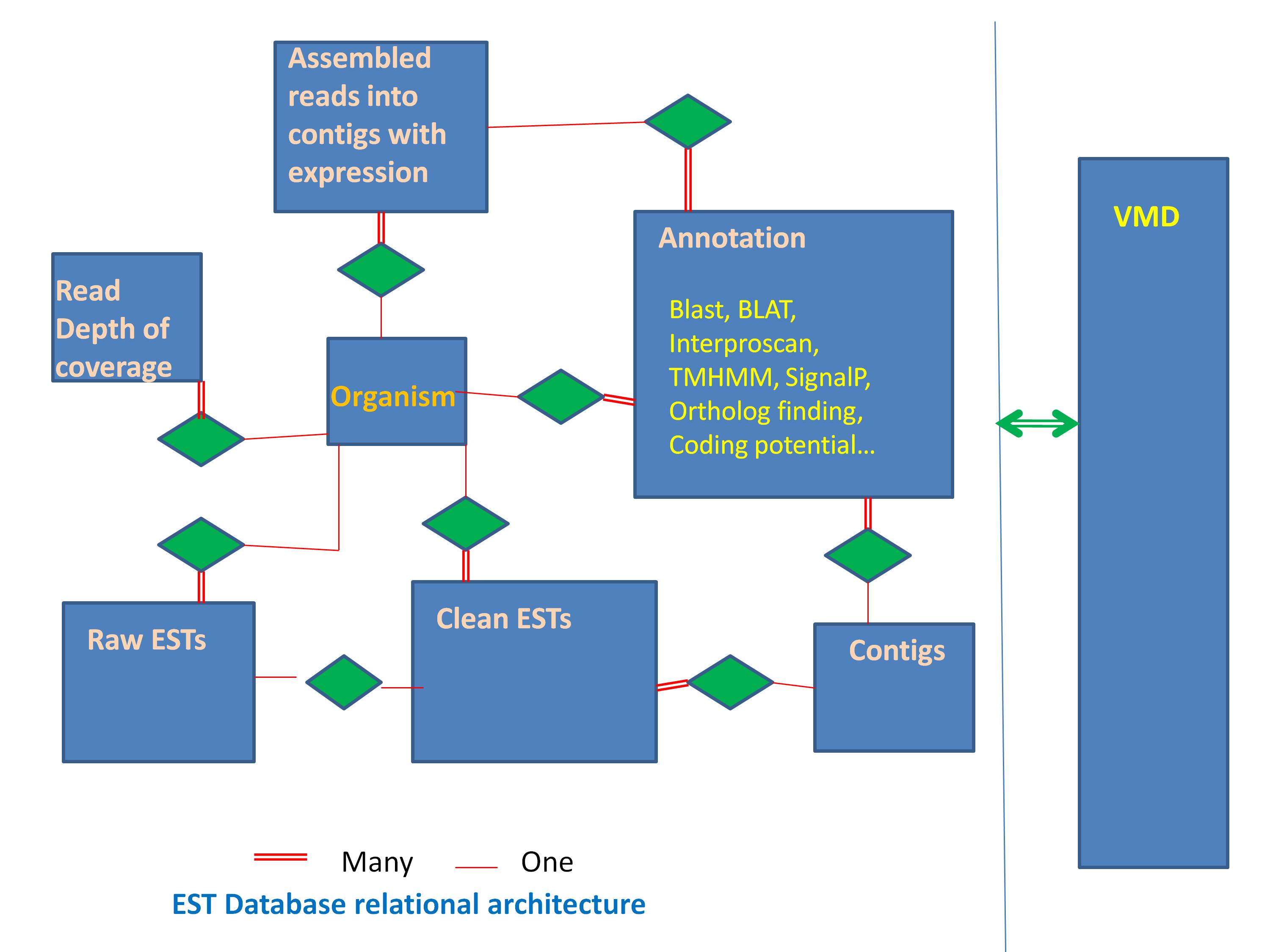

Supplement: Additional file 2 — Figure S2. Entity-Relationship diagram of OTD. [file 1471-2164-13-303-S2.jpeg]
