# Supplementary material for: Oomycete transcriptomics database: A resource for oomycete transcriptomes
Source: BMC Genomics. 2012 Jul 6;13:303. doi: 10.1186/1471-2164-13-303 (PMC3542173; doi:10.1186/1471-2164-13-303)
Supplement: Additional file 3 — Documentation on how to use the oomycete transcriptomics database. [file 1471-2164-13-303-S3.docx]

**Documentation on How To use the Oomycete Transcriptomics Database:**

To begin with: if you have a sequence, for example from the *P.sojae* V1 assembly, and would like to ascribe some biological meaning to it, begin with the [blast page](http://www.eumicrobedb.org/cgi-bin/ESTBlast/cgi-bin/nph-blast.pl). Paste your sequence into the specified area, choose your blast parameters, then select a database (may be Transcript Assembly from *P. sojae* Infected sample V1.0). The output provides a detailed graphical page listing the hits in descending order [Fig – 1]


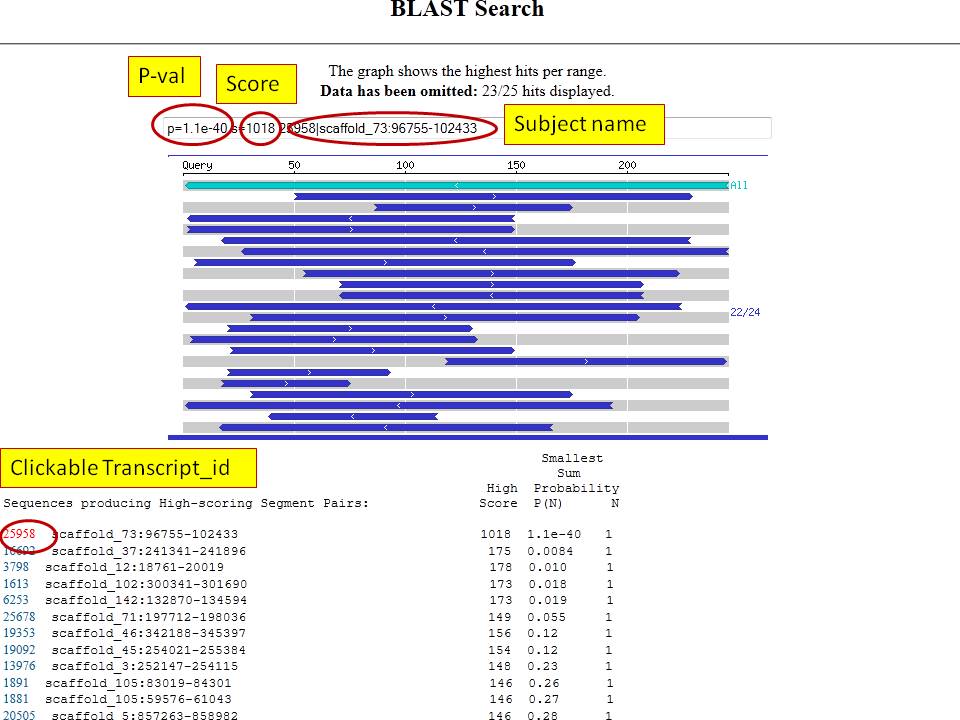


Fig -1 : Blast output page.

Next, click on the assembled_transcript_id (circled); this will take you to the assembled transcript page derived from transcripts of *P. sojae* infected materials against assembly version 1 [Fig – 2]. The location of the assembly on the genome appears just next to the transcript_id on the blast page.

The transcript assembly page is loaded with a lot of information. The name of the library (e.g. “WI” here means ‘with infection’) from where the transcript has been assembled. The scaffold_location on the first row of the output page links to the browser and is fully clickable [Fig-3]. There is a link for running on-the-fly blast against the Genbank NR database and the output is displayed as a new page [Fig 2A].


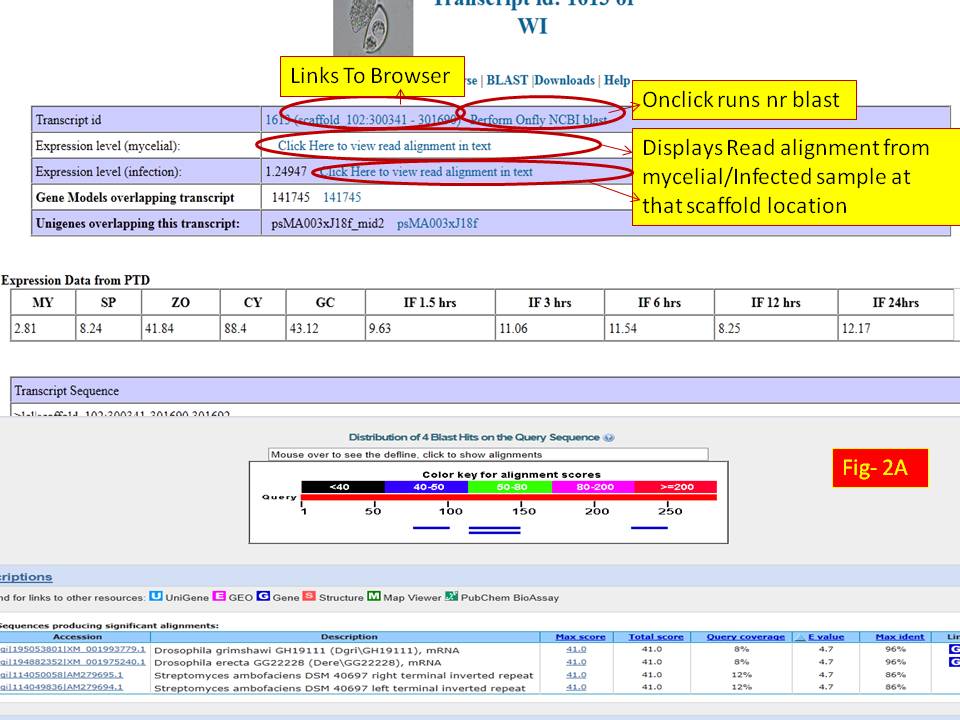


Fig-2 and 2A: Main transcript page and onfly blast output.

The browse link opens to a browser page, where tracks corresponding to infected samples, mycelia samples, unigene alignments of related organisms are displayed. In Fig-3A, there are 5 tracks. The first track corresponds to the predicted gene models. The second plot depicts the read depth-of-coverage plot where the orange colored area is the depth coverage for infection samples and the blue colored graphs are from mycelial samples. The next track is the assembled mycelia transcript track followed by the assembled infection transcript track. The tracks following that are the unigene alignment tracks. These tracks are color coded on the basis of their alignment quality. If the number of query gaps are greater and the alignment is not contiguous, then it is categorized as a poorer alignment.

In this given screen shot the links from transcript page open to the browser [Fig 3A]. Extending the view on each side reveals [Fig 3B] the predicted gene model to be shorter at the 5’ end than the assembled transcript. The black transcript tracks indicate a low expression level. The EST alignment track indicates there is very good EST evidence in this region, but the expression level still remains low.


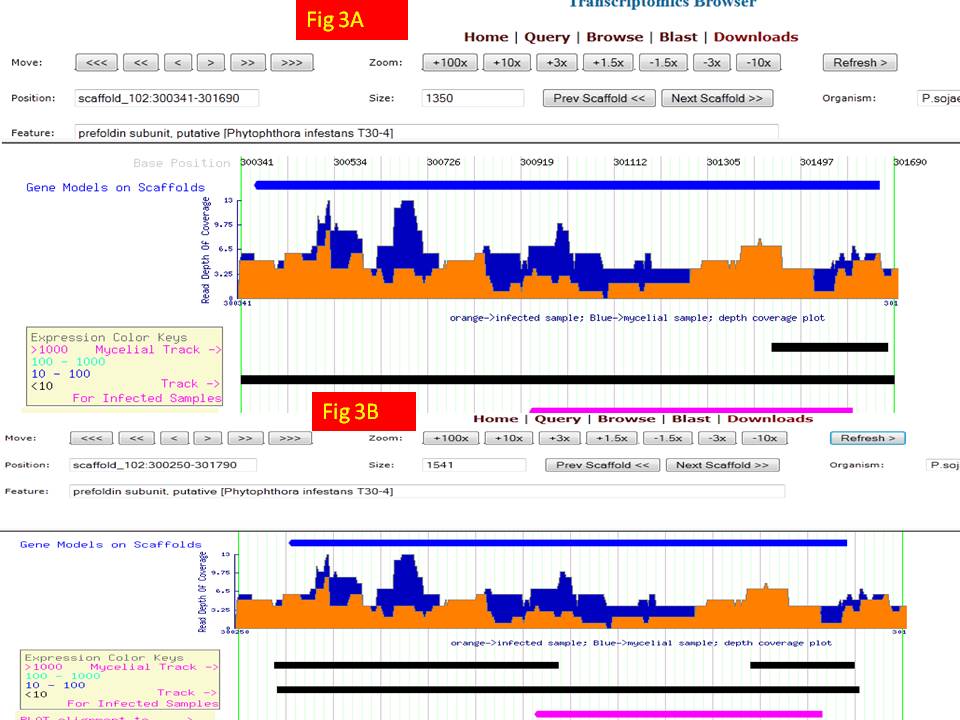


**Using Query Page: [Fig - 4]**

The query page is classified into 2 distinct types:

1. Query by assembled transcripts
2. Query by ESTs/Unigenes.

**Query By Assembled Transcripts:**

This category is again sub-divided into 2 types:

1. Query by expression range (as calculated by FPKM value)
2. Query by fold-change between infection and non-infection samples

Query by expression range takes a range value (e.g; with >, <, - operators). If one wants to retrieve highly expressed genes in *P. sojae* mycelia, just enter “>100”. A list will be retrieved detailing the expression ranges. Each of the entries (transcript_ids) are clickable and link to the transcript page [Fig 2]. Also, one could find the genes showing a fold change of say 10 between infection and non-infection conditions, then a similar list is displayed with transcript_id links to individual pages.


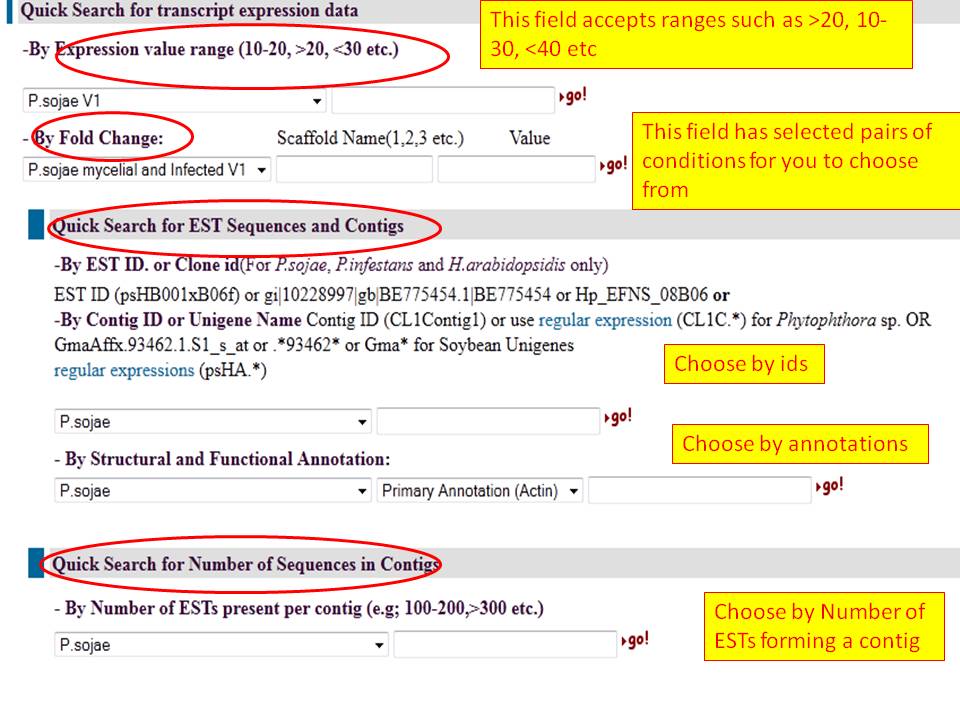


**Fig - 4: Details of Query page**

**Query By ESTs/Unigenes:**

There are a number of organisms listed in this database that have EST and assembly information. For *P. sojae* the processes leading to adaptor trimming and removal of poor quality regions are also recorded in the database. One can query ESTs by their name or by a wild card. Several naming conventions exist for different organisms such as:

1. P. sojae ESTs begin with ‘ps’, *P. infestans* unigenes from our center begin with ‘pi’. P. infestans ESTs from genbank have a prefix ‘gi|’ and Hyaloperonospora ESTs begin with Hp and soybean ESTs begin with ‘Gma’. One can use wild card search to query the EST sequences from this database. For all ESTs from HA library (Infected Soybean mycelium: details in the metadata info links available from the main page), search by psHA.*.
2. All contigs begin with abbreviation ‘CL’. So choosing an organism and entering CL1C* will retrieve all the contigs beginning with “CL1C” [Fig 6].

The output pages from the EST and Unigenes pages load into the EST detail page and unigene detail page, respectively.

**EST detail Page [Fig 5A, 5B]:**

**
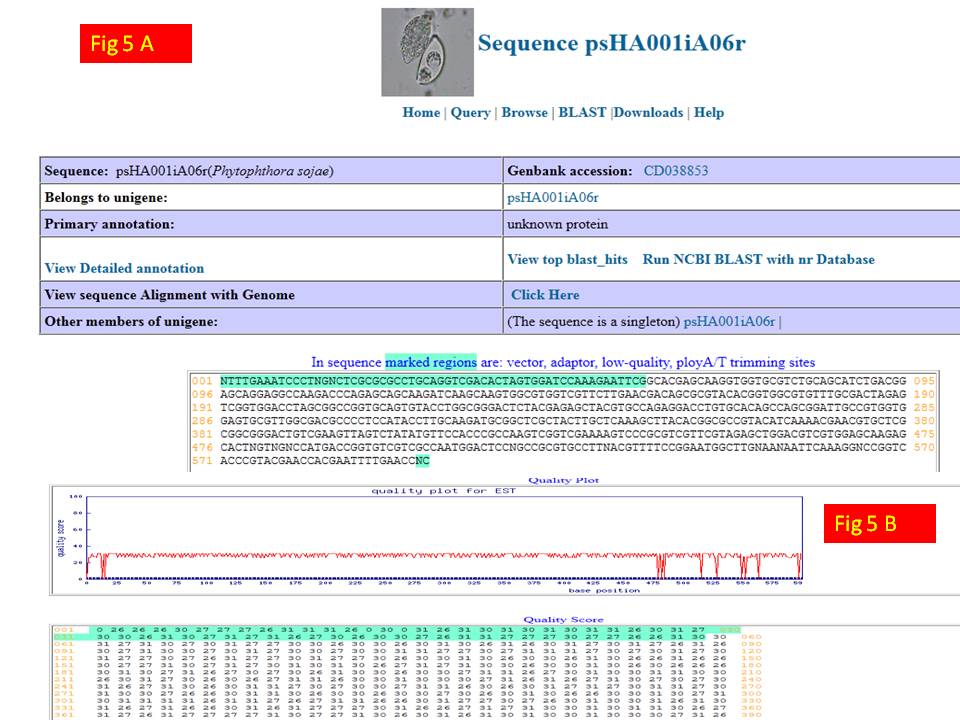
**

The EST detail page also lists a large amount of information such as Quality trimming and adaptor trimming information, . the number of other ESTs that assemble together with this EST to form a contig, etc. One can also run on-the-fly blast from this page. On-the-fly genome sequence alignment via BLAT is also available through this page.

**Main Contig Page:**

The main contig page has a large amount of information on the contigs including contig assembly, contig quality and overlapping gene models [Fig 6A, 6B]. Users can run on-the-fly BLAT against the genome assembly by clicking on the ‘Run alignment against genome’ link [Fig 6 C].


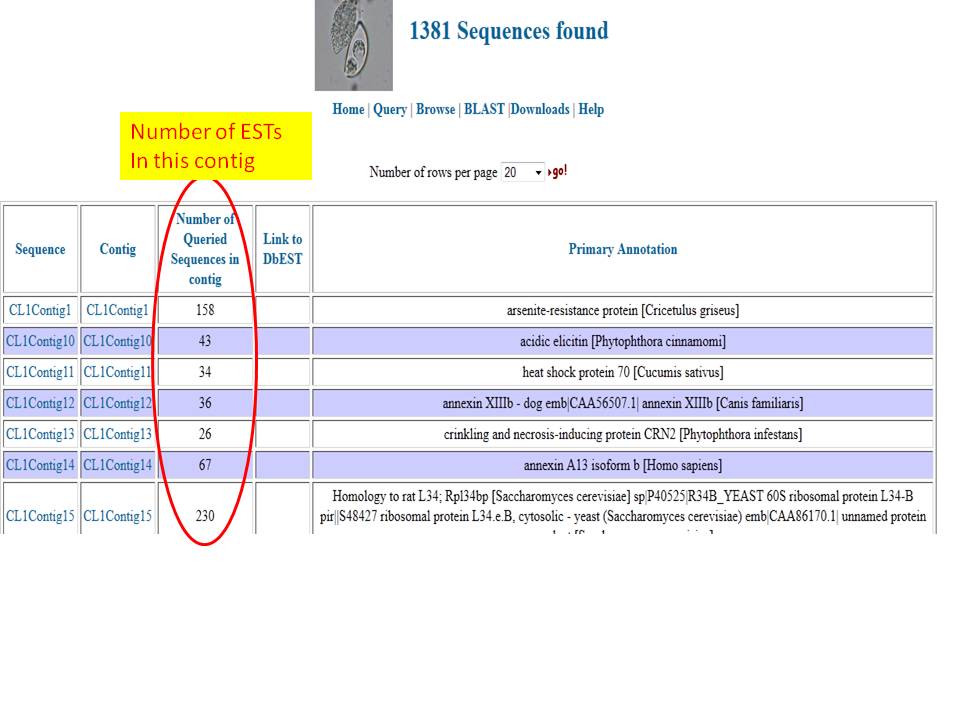


Fig 6: Contig wild card search page


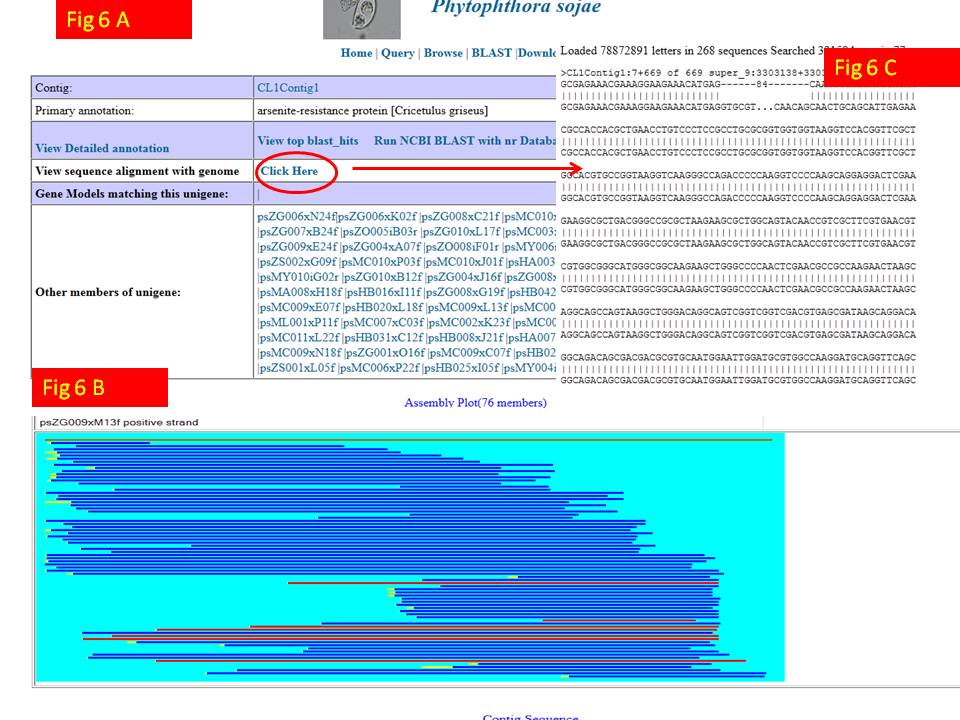


Fig 6A,B,C : details of Contig Assembly page.

The end product of a unigene is stored in a main contig annotation page. This page has blast, interproscan, TMHMM, SignalP annotations for individual unigenes [Fig 7].


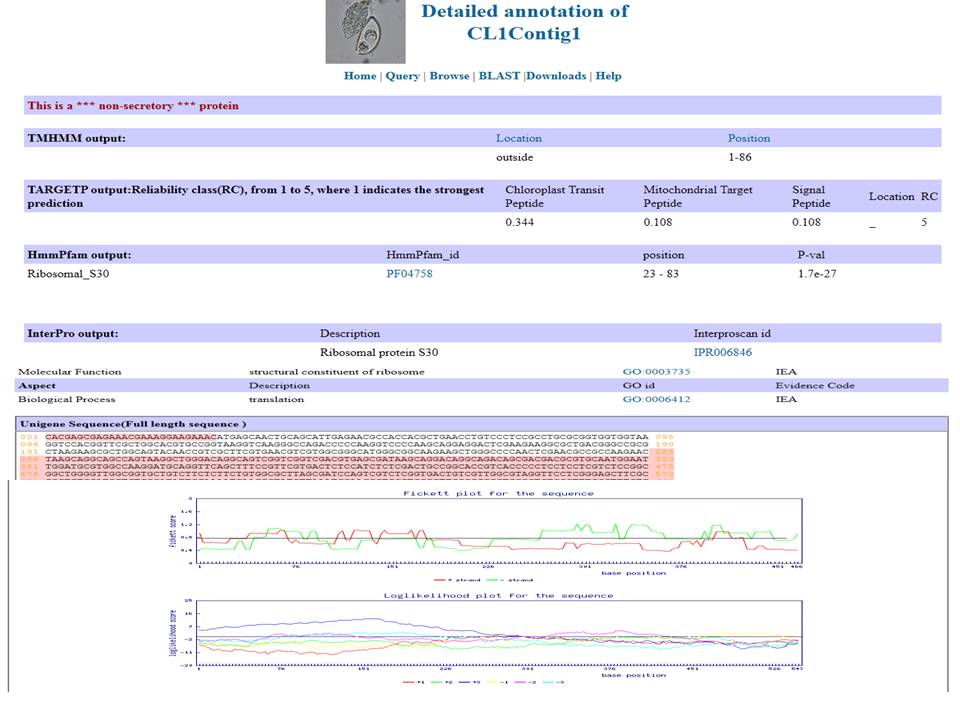


Fig 7: Depicting the main annotation page.

A valuable feature of this database is the SNP and alignment viewer for assembled transcripts. From the main transcript page [Fig 2], one can click to get information on detailed read assembly. The genome reference sequence remains on top and static and follows the window down as one scrolls down thus making the visual effect more clear. This page figuratively describes the details of read assembly and one can compare read sequences between two different conditions such as: Infected samples and mycelia samples of *P. sojae* V1.0 and so on [Fig 8]


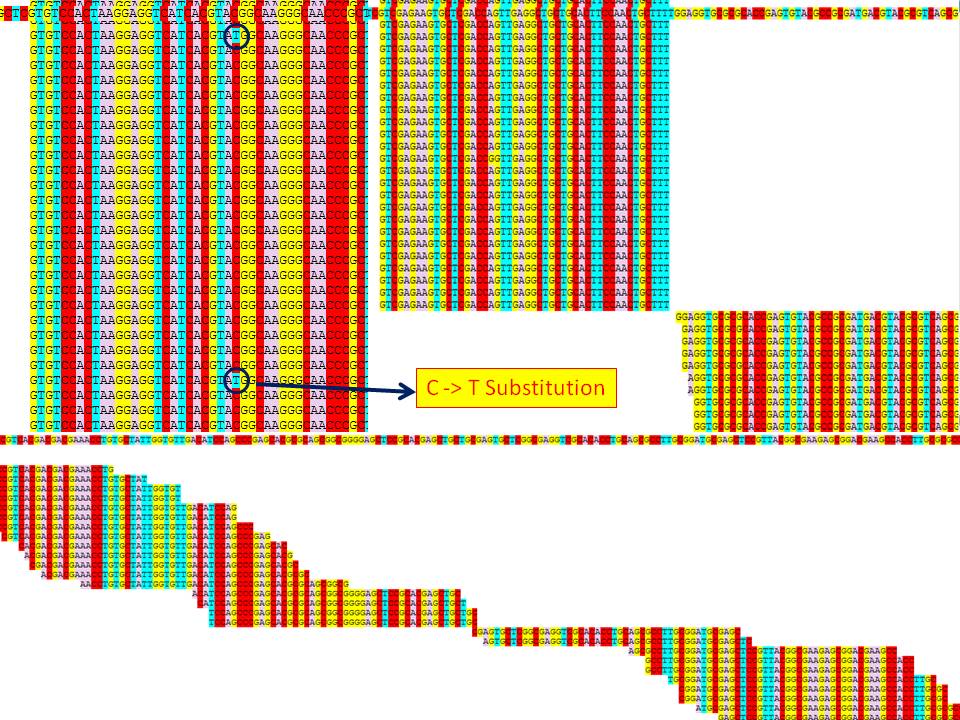


Fig 8: Reads assembly/Alignment against genome reference

**Miscellaneous Features:**

Apart from data from different organisms, metadata, cluster, assembly statistics etc. are available from the main transcriptomics database home page.

There is also a download page that has a lot of curated data available for download. Any data type not available for download can be requested through the request form. In addition to a FAQ page, a help page is also set up for quick reference.
